# Interaction of *Listeria monocytogenes* with the human choroid plexus endothelium in vitro: impact on invasion of the epithelium

**DOI:** 10.1007/s13577-026-01419-8

**Published:** 2026-07-10

**Authors:** Carolin Stump-Guthier, Sandrin Schulze, Walter Muranyi, Mobarak Abu Mraheil, Torsten Hain, Michael Hagmann, Hiroshi Ishikawa, Horst Schroten, Christian Schwerk, Stefan Weichert

**Affiliations:** 1 Pediatric Infectious Diseases, Department of Pediatrics, Medical Faculty Mannheim, Heidelberg University, 68167 Mannheim, Germany; 2 Institute of Medical Microbiology, German Center for Infection Research (DZIF), Partner Site Giessen-Marburg-Langen, Justus-Liebig University Giessen, 35392 Giessen, Germany; 3 Department of Medical Statistics, Biomathematics and Information Processing, Medical Faculty Mannheim, Heidelberg University, 68167 Mannheim, Germany; 4 Laboratory of Clinical Regenerative Medicine, Department of Neurosurgery, Faculty of Medicine, University of Tsukuba, 1-1-1 Tennodai, Tsukuba, Ibaraki 305-8575 Japan

**Keywords:** Blood–cerebrospinal fluid barrier, Choroid plexus, Endothelium, Epithelium, Host–pathogen interaction, *Listeria monocytogenes*

## Abstract

In the central nervous system (CNS), the highly vascularized choroid plexus (CP) produces the cerebrospinal fluid (CSF) and also acts as a blood-CSF barrier (BCSFB). Due to its location in the ventricular system of the brain, the CP can be used by pathogens such as bacteria to enter the CNS by crossing from the blood into the brain. This process, which can lead to meningitis, encephalitis and meningoencephalitis, is usually prevented by the BCSFB. Since barrier function is thought to be mainly executed by the CP epithelium, whose cells are connected to each other by dense tight junction (TJ) strands, in vitro studies of bacterial pathogens interacting with the BCSFB have routinely been performed using CP epithelial cells only. Recent data have shown that the CP endothelium can also contribute to barrier function at the BCSFB. Here, we used immortalized human CP endothelial cells (iHCPEnC) to investigate the interaction of the CP endothelium with the bacterial pathogen *Listeria monocytogenes* (*Lm*). Although *Lm* adhere to iHCPEnC, the endothelial cells are only weakly invaded by *Lm*, but readily transmigrated, in contrast to a CP epithelial cell line (HIBCPP cells). In a two-cell type model of the CP *Lm* transmigrated across iHCPEnC and invaded into HIBCPP cells, indicating that the CP endothelium does not pose a particularly strong barrier to *Lm* when attempting to overcome the BCSFB. Epithelial invasion was reduced compared to a model consisting of only HIBCPP cells, suggesting a regulatory impact of the CP endothelium.

**Supplementary Information:**

The online version contains supplementary material available at 10.1007/s13577-026-01419-8.

## Introduction

The choroid plexus (CP), located in the brain ventricles, consists of a conglomerate of several cell types, including the fenestrated endothelium and the epithelial cells. Whereas the epithelial cells are connected by tight junctions (TJ) to execute barrier function, the endothelial cells are rather leaky, and present properties different from those of the barrier-forming endothelial cells of the blood–brain barrier (BBB) [1–3].

*Listeria monocytogenes* (*Lm*), a gram-positive foodborne, facultative intracellular pathogen, can cause severe complications in newborns and immunocompromised individuals. After oral uptake, *Lm* is able to cross the epithelial barrier of the gut and to enter the bloodstream. Via circulation it can encounter, invade and overcome other host barriers, such as the BBB and the BCSFB, leading to meningitis or meningoencephalitis [4, 5]. During invasion of host cells, binding of listerial virulence factors to cellular surface proteins is involved. Important engagements have been described for the listerial internalins (Inl) InlA, InlB, and InlF with E-Cadherin, receptor Met (Met) and Vimentin, respectively [6].

For studying bacterial interactions at the CP, an epithelial model based on human CP papilloma (HIBCPP) cells was used extensively [7–10]. We could show that the HIBCPP cells present a high transepithelial electrical resistance (TEER) and therefore suit well as a barrier-forming model for mimicking a blood-cerebrospinal fluid (CSF) barrier (BCSFB) in vitro [10].

Using this model, we have analyzed the interaction of *Lm* with the CP epithelium [11–13]. *Lm* invades HIBCPP cells in a polar fashion from the basolateral side, a process that interdependently requires InlA and InlB [12].

To study cellular processes at the CP in more detail, sophisticated in vitro models that incorporate more than one cellular component are required [14, 15]. Recently we generated an immortalized endothelial cell line (iHCPEnC) which is able to support the epithelial HIBCPP cells when applied in a co-culture model [16, 17]. Together, both cell lines form an in vivo near-conglomerate that represents the human CP more realistically than HIBCPP cells alone.

Here, we investigate in vitro the interaction of CP endothelial cells with a bacterial pathogen, i.e., *Lm*, and delineate the impact of the endothelium on invasion of the epithelium at the CP.

## Materials and methods

### Bacterial and cell culture

For infection experiments the *Lm* 1/2a strains EGDe and its isogenic deletion mutant InlF were used [13, 18]. Bacterial culture was performed as described previously [11].

Experiments with iHCPEnC were performed as described before [16]. In brief, iHCPEnC were seeded onto Attachment Factor (Cell Systems, Kirkland, USA) coated 24-well coverslips in a density of 5 × 10^5^ cells/well in complete classic endothelial medium (Cell Systems, Kirkland, USA). After 5–6 days medium was changed to 1% HIBCPP cell culture medium (DMEM/HAMS F12 medium (Gibco, Darmstadt, Germany), supplemented with 1% fetal calf serum (FCS) and 5 µg Insulin/ml). Infection experiments were performed the day after. A multiplicity of infection (MOI) of 10 was used.

Co-culture was performed as described before [16, 17]. In brief, 8 × 10^4^ HIBCPP cells were seeded upside down onto a 24-well filter insert with 3 µm pores (#662631, Greiner Bio One, Frickenhausen, Germany) establishing an inverted cell culture. 5–6 days after seeding of the HIBCPP cells, TEER measurements are performed using a chopstick electrode (Millipore, Schwalbach, Germany) [10]. When the TEER reached values around 100 Ohm * cm^2^, the medium was changed to 1% HIBCPP cell culture medium and 4 × 10^5^ iHCPEnC were seeded into the upper well of the filter insert. The cells were used for experiments when TEER values of the HIBCPP cells cultured alone raised above 200 Ohm * cm^2^ and the TEER values of the co-cultures displayed values 1.5-fold higher compared to HIBCPP cells alone.

The culture of human brain microvascular endothelial cells (HBMEC) and hCMEC/D3 was performed as previously described [19, 20].

### RT-PCR

Conventional RT-PCR was performed as described before [10]. In brief, RNA was extracted with the help of the RNeasy Micro Kit (Qiagen, Hilden, Germany) followed by cDNA synthesis (Santa Clara, California, USA). PCR reaction was carried out according to the manufacturer’s protocol (Qiagen, Hilden, Germany) and visualized with the help of an 1.5% Agarose gel using Ethidium bromide (primers are listed in Table 1).

**Table 1:** Primers used for RT-PCR

	Forward	Reverse	Amplicon size (bp)
CDH1	GAGAACGCATTGCCACATACAC	GAGCACCTTCCATGACAGACCC	162
MET	ATCTTGGGACATCAGAGGGT	TCGTGATCTTCTTCCCAGTGA	174
VIM	AGAGAGAGGAAGCCGAAAAC	TGGATTTCCTCTTCGTGGAGTT	145
CDH5	TCACCTT CTGCGAGGATATGG	GAGTTGAGC ACCGACACATC	244
GAPDH	TGTTGCCATCAATGACCCCTT	CTCCACGACGTACTCAGCG	202

### Western blot

Western blot analysis was performed as mentioned before [11]. In brief, whole protein lysates were collected using a modified RIPA lysis buffer (1 × RIPA lysis buffer, 50 mM NaF, 1 mM Na_3_VO_4_, protease inhibitor cocktail). 10 µg protein lysate were separated with a 4–12% Bis–Tris gel (Thermo Fisher Scientific Inc., Waltham, Massachusetts, USA) and transferred to a nitrocellulose membrane (Biorad, Hercules, California, USA). Primary antibodies were applied o/n at 4 °C, followed by secondary antibodies for 1 h (antibodies listed in Table 2). The proteins were visualized by Radiance plus Chemiluminescence (Azure Biosystems, Dublin, California, USA).

**Table 2:** Antibodies used for Western blotting

Primary antibody	Dilution	Source	Company	Art. no
CDH1	1:1000	Mouse	BD, Franklin Lakes, New Jersey, USA	610182
Met	1:1000	Rabbit	Cell Signaling, Danvers, Massachusetts, USA	8198
Vim	1:1000	Chicken	Biolegend, San Diego, California, USA	919101
GapDH	1:1000	Rabbit	Cell Signaling, Danvers, Massachusetts, USA	2118
CDH5	1:1000	Rabbit	Cell Signaling, Danvers, Massachusetts, USA	2500
Actin	1:1000	Mouse	Sigma, Deisenhofen, Germany	A5541
Secondary antibody	Dilution	Source	Company	Art. no
Anti-rabbit-HRP	1:5000	Donkey	Millipore, Schwalbach, Germany	AP182
Anti-mouse-HRP	1:5000	Donkey	Millipore, Schwalbach, Germany	AP192
Anti-chicken-HRP	1:5000	Donkey	Millipore, Schwalbach, Germany	AP194P

### Double immunofluorescent staining to visualize invaded and adhered *Lm*

Double immunofluorescence was performed as described before [12]. In brief, after *Lm* incubation the cells were washed, blocked and incubated with the first antibody (rabbit anti-*L. monocytogenes*; 1:500; Meridian Life Science, Memphis, TN, USA) to stain extracellular bacteria, followed by subsequent washing and fixation steps. The first antibody was labeled with a secondary antibody (Alexa Fluor 594 (red) chicken-anti-rabbit antibody; 1:250; Thermo Fisher Scientific Inc., Waltham, Massachusetts, USA), followed by washings and a permeabilization step. The first antibody was added a second time to stain both extra- and intracellular bacteria, followed by addition of a second secondary antibody (Alexa Fluor 488 (green) donkey-anti-rabbit antibody; 1:500; Thermo Fisher Scientific Inc., Waltham, Massachusetts, USA). To stain the cytoskeleton and the nuclei, Phalloidin Alexa Fluor 660 (1:250; Thermo Fisher Scientific Inc., Waltham, Massachusetts, USA) and 40–6-diamidino2-phenylindole dihydrochloride (DAPI) (1:50,000) were applied.

Evaluation of adhered and invaded bacteria was performed as described before [10].

### Statistical analysis

Data from transmigration and invasion experiments were analyzed using linear mixed-effects models, accounting for heteroscedasticity and clustering within replication runs. For transmigration assays, the analysis was restricted to the HIBCPP condition; fixed effects included incubation time (for the TEER outcome) and membrane type (filter membrane ± cells; for the transmigration outcome). For invasion experiments, culture model was included as a fixed effect. Statistical significance was defined at *p* < 0.05. To maintain the family-wise error rate, *p* values for all post-hoc comparisons were adjusted using the single-step method via the multcomp package in R.

## Results

### Expression of surface receptors for *Lm* by iHCPEnC

**Fig. 1:**
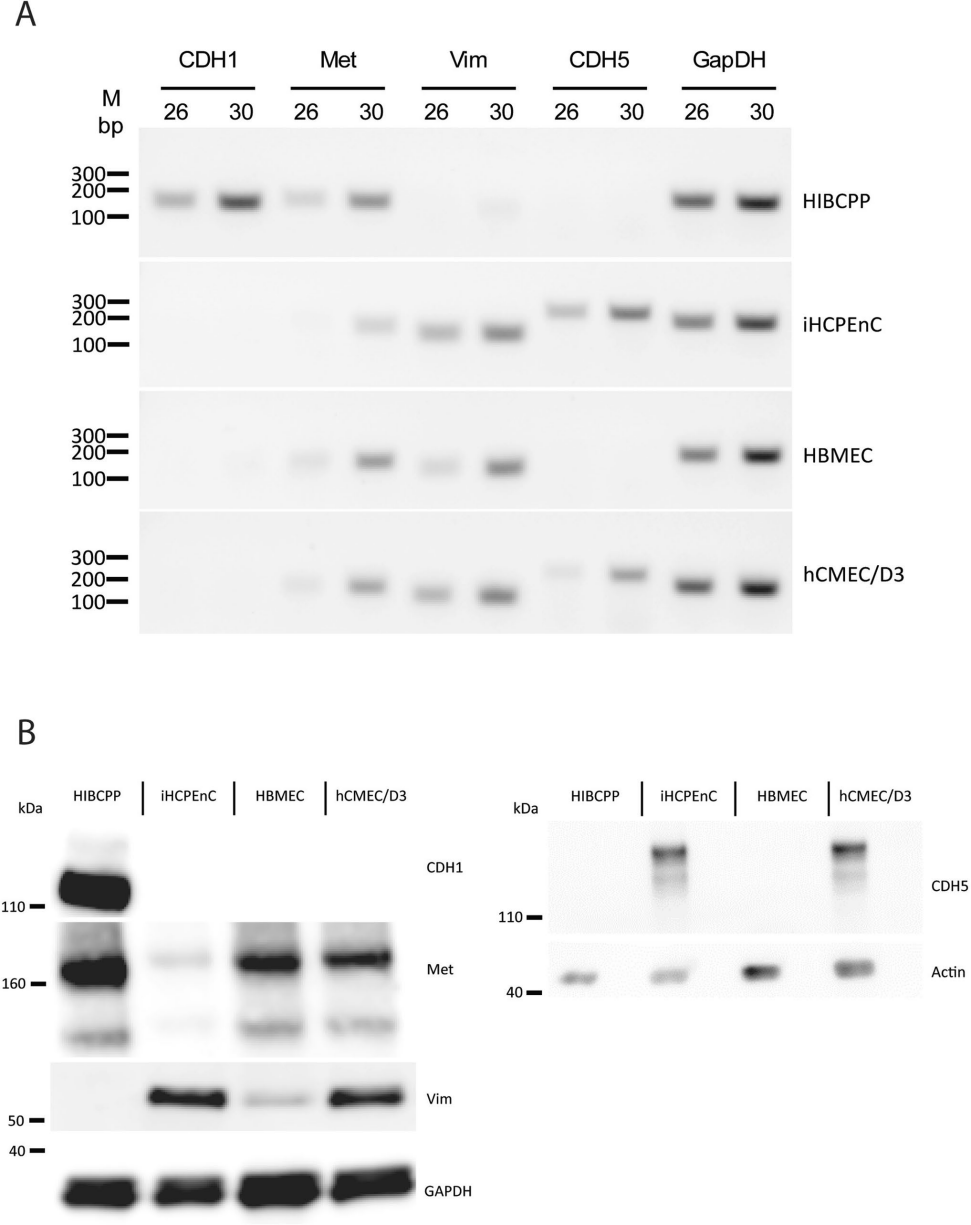
Analysis of the expression of the surface receptors CDH1, Met, Vimentin (Vim) and CDH5 in iHCPEnC, HIBCPP, HBMEC, and hCMEC/D3. **A** Transcription levels of genes of interest, as determined by RT-PCR. Gene names are indicated on top. The amplificates were separated on 1.5% agarose gels, size markers are indicated on the left. **B** Protein expression levels of proteins of interest detected by Western blot. Protein names are indicated on the right. The separation of the proteins was performed using a 4–12% Bis–Tris MOPS buffered gels. Presented are representative data selected from experiments performed in triplicates

iHCPEnC were characterized for genes that serve as potential receptors for virulence factors of *Lm* by conventional RT-PCR (Fig. 1A). Expression of CDH1, Met, and Vimentin in iHCPEnC was compared to their expression in HIBCPP cells and in the human brain microvascular endothelial cell lines HBMEC and hCMEC/D3. CDH1 was only expressed in the epithelial HIBCPP cells, whereas Met was expressed in all cell lines. Vimentin was mainly detected in the endothelial cell lines iHCPEnC, HBMEC and hCMEC/D3. VE-Cadherin (CDH5) expression was analyzed as a control and was found in iHCPEnC and hCMEC/D3, but not in HBMEC and HIBCPP cells.

Expression of CDH1, Met, Vimentin and CDH5 was confirmed on protein level by Western blotting and corresponded to their expression on RNA level (Fig. 1B). HBMEC seem to have lost expression of CDH5. Very low expression levels of CDH5 in HBMEC in comparison to hCMEC/D3 have been described before [21].

### Transmigration of *Lm EGDe* across iHCPEnC and HIBCPP cells

**Fig. 2:**
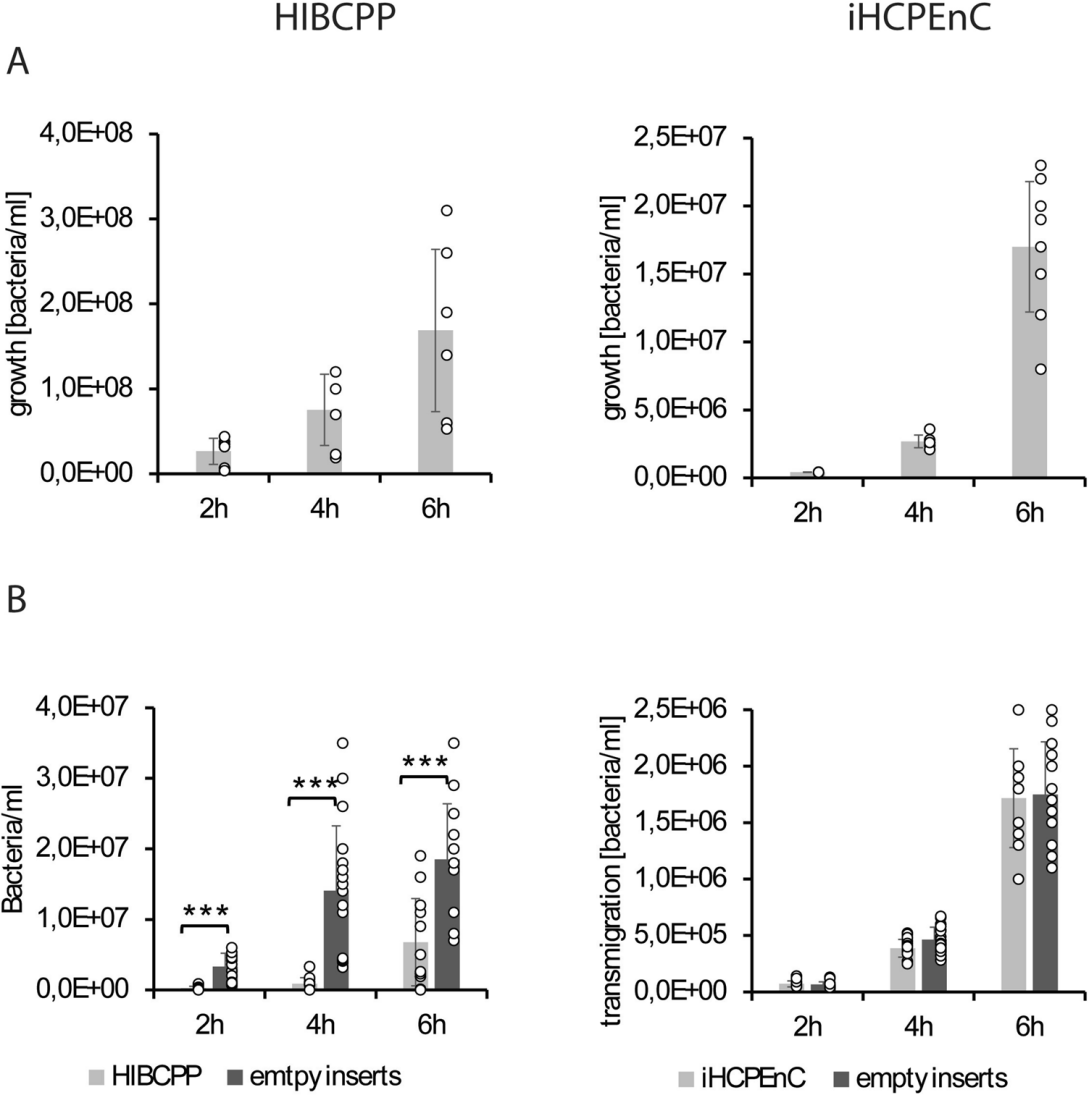
Analysis of transmigration behavior of *Lm* through HIBCPP and iHCPEnC. Data is shown as mean ± standard deviation. **A** Growth curves for *Lm* EGDe run in parallel to the transmigration experiments. **B** Bacterial transmigration of *Lm* EGDe over HIBCPP cells in inverted culture or iHCPEnC in standard culture for 2, 4 and 6 h. Cell culture filter inserts with HIBCPP cells or iHCPEnC, respectively, are compared to inserts without cells (empty inserts). In both conditions MOI 10, according to the number of cells present in the filter membrane, was applied. All experiments were performed at least three times in triplicates. ***highly significant (*p* < 0.001)

Transmigration experiments of *Lm* were carried out to investigate the extent to which HIBCPP cells and iHCPEnC pose an obstacle for the passage of bacteria (Fig. 2). HIBCPP cells present a strong barrier function that is significantly challenged after 4/6h of incubation with *Lm* (Fig. S1), confirming previous results [12], whereas iHCPEnC display no barrier function as expected [16]. Figure 2A shows growth curves of *Lm* performed during the transmigration assays. For infection of HIBCPP cells, higher amounts of bacteria are required in contrast to iHCPEnC to reach an MOI 10. Whereas HIBCPP cells significantly reduce transmigration, iHCPEnC do not inhibit the transmigration of *Lm* (Fig. 2B).

### Infection of iHCPEnC with *Lm*

**Fig. 3:**
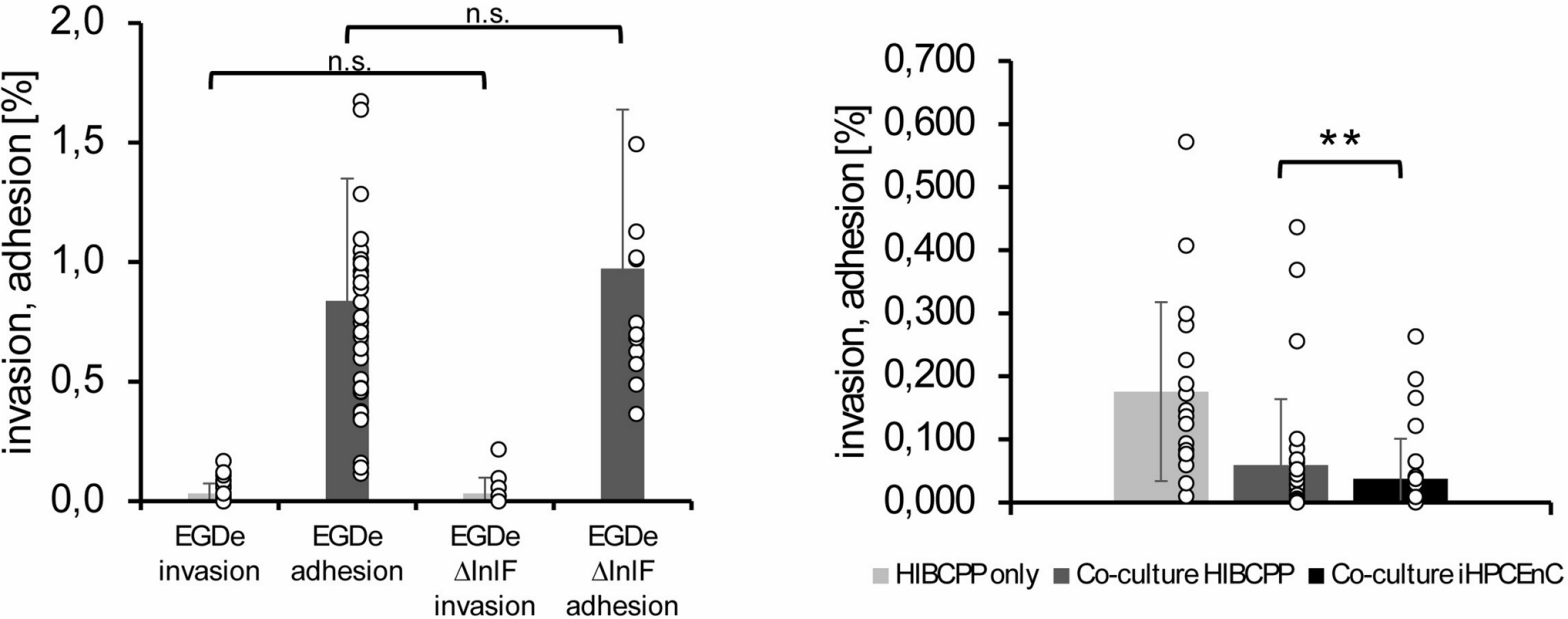
Infection of iHCPEnC, HIBCPP only or HIBCPP co-cultured with iHCPEnC analyzed by double immunofluorescence. Each experiment was repeated at least three times with at least two replicates. Invasion and adhesion were counted after 4 h. Data are shown as mean ± standard deviation. **A** Comparison of invasion (dark gray bars) and adhesion (light gray bars) of *Lm* EGDe (MOI10) with *Lm* EGDe ΔInlF (MOI10) in iHPCEnC cultured on coverslips. **B** Invasion data of *Lm* EGDe in HIBCPP cells only as well as HIBCPP cells and iHPCEnC grown together in the two-cell type culture model. Cultures were infected with an MOI10 for iHCPEnC, which corresponded to an MOI1 for HIBCPP cells. HIBCPP cell monocultures were infected with an MOI1 as well. n.s. = not significant (*p* > 0.05); **very significant (*p* < 0.01)

We monitored to which extent *Lm* adhere to or invade into iHCPEnC (Fig. 3A). After infection with an MOI 10 for 4 h, we found around 0.84% of *Lm* EGDe adhered to iHCPEnC. We could only observe a very low invasion rate of 0.03%. An InlF deletion mutant (EGDe ΔInlF) was analyzed in parallel, as iHPCEnC express Vimentin. Invasion as well as adhesion rates of EGDe ΔInlF are comparable to EGDe, which represents the wild type strain.

### Infection of the two-cell type culture system with *Lm*

Finally, we investigated invasion rates of *Lm* EGDe in the co-culture model, build-up of endothelial- and epithelial cells of the CP. The experiments were carried out with an MOI 10 for iHCPEnC which turns out for HIBCPP cells as an MOI 1. In parallel we compared the infection of HIBCPP cells only, here with an MOI 1 as well. Invasion into iHCPEnC cultured in the co-culture system (Fig. 3B) stayed comparable to invasion into iHCPEnC alone cultured on coverslips (Fig. 3A). In contrast, invasion of *Lm* into HIBCPP cells co-cultured with iHCPEnC was significantly reduced compared to HIBCPP cells only (Fig. 3B). TEER values of HIBCPP cell cultures, alone and in the co-culture model, were stable during the course of the experiments (Fig. S2).

## Discussion

By analyzing host cell surface receptors as potential targets for virulence factors of *Lm*, we determined that iHCPEnC express Vimentin and some Met. As expected for endothelial cells, no CDH1 could be detected. Despite the fact that we observed a high amount of *Lm* adhered to iHCPEnC (around 0.84%), invasion rates were rather low (around 0.03%). Although Met and Vimentin are expressed, the lack of E-Cadherin is a possible explanation for this low invasion. This recapitulates the strong drop of invasion of *Lm* into HIBCPP cells, which express both E-Cadherin and Met, when InlA, the interactor for E-Cadherin, is deleted from *Lm* [12]. Gosh and colleagues have proposed that during colonization of the brain by *Lm* InlF and Vimentin are involved [22]. In our experiments, deletion of InlF from *Lm* had no impact on invasion of iHCPEnC, and in a previous study [13] we made the same observation when listerial invasion of HBMEC, which also express Vimentin, was investigated. As also discussed there, clarification would require further investigation, including research on differences between various human microvascular endothelial cell lines and on potential roles of Rho-associated protein kinases [13].Although we have not shown direct evidence for involvement of InlB-Met interactions, our data at least suggest that the main route of invasion into iHCPEnC is mediated by InlB and Met and not via InlF and Vimentin.

iHCPEnC enhance the TEER of HIBCPP cells when they are co-cultured, but they do not present a significant barrier function by themselves [16]. In accordance, *Lm* could easily transmigrate cell layers of iHCPEnC cultured on cell culture filter inserts, whereas HIBCPP cells significantly reduced transmigration. These data indicate that iHCPEnC do not present a strong obstacle to *Lm* entering the CNS across the BCSFB.

Although iHCPEnC do not present an obstacle, reduced invasion of *Lm* into HIBCPP cells was found in the two-cell type culture model as compared to HIBCPP cells only. The observed adhesion of *Lm* to iHCPEnC could limit invasion of HIBCPP cells to a certain extent. Still, the adhesion rate of around 1% should not have a major impact, and transmigration rates of *Lm* in presence of iHCPEnC were not reduced compared to transmigration through empty filter inserts. Additionally, accessibility of bacteria to the basolateral surface of HIBCPP cells, which is limited to the pores in the filters of the cell culture inserts, controls invasion rates of *Lm* into HIBCPP cells. It is conceivable that in the two-cell type model the iHCPEnC layer impacts access of *Lm* to the filter pores, but, again, iHCPEnC did not inhibit transmigration of *Lm*.

The most interesting, and possibly plausible, mechanism would involve interplay between endothelial and epithelial components in the two-cell type model. Reduced invasion may be correlated with the increased barrier function of the two-cell type culture model, in the end resulting in less bacteria being able to infect the CNS. This mechanism could involve regulation of cell adhesion molecules in HIBCPP cells. Alternatively, iHCPEnC may act on the expression of cellular factors of HIBCPP cells, including surface receptors, required during invasion by *Lm*. The exact processes involved in this mechanism would require further investigation.

## Supplementary Information

Below is the link to the electronic supplementary material.

Supplementary file1 (PDF 425 KB)

Supplementary file2 (PDF 424 KB)

## Data Availability

Not applicable.
