# Supplementary material for: Interaction of Listeria monocytogenes with the human choroid plexus endothelium in vitro: impact on invasion of the epithelium
Source: Hum Cell. 2026 Jul 10;39(7):104. doi: 10.1007/s13577-026-01419-8 (PMC13354639; doi:10.1007/s13577-026-01419-8)
Supplement: Supplementary file 1 — Supplementary file1 (PDF 425 KB) [file 13577_2026_1419_MOESM1_ESM.pdf]

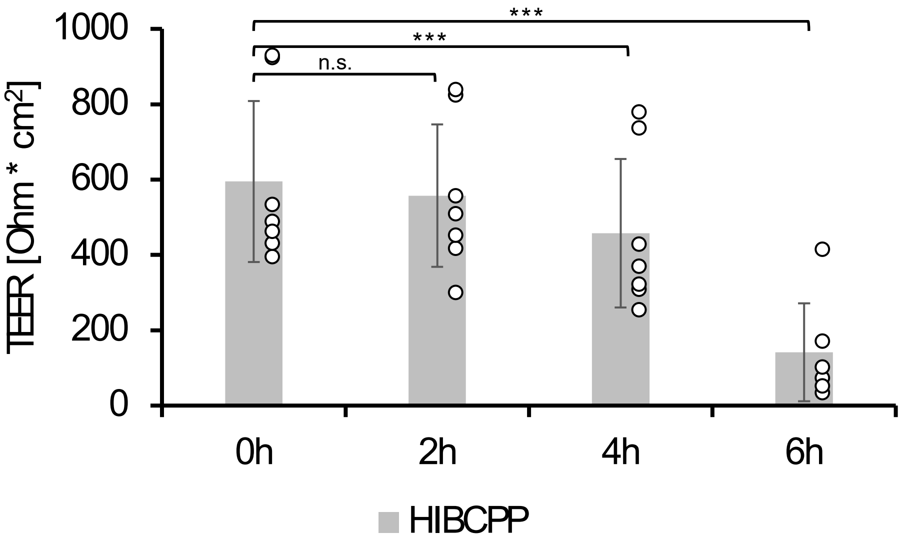

Fig. S1 TEER values of HIBCPP cells cultured in inverted culture system at the time of infection (0h) and during the experiments (2, 4, 6h). All experiments were performed at least three times in duplicates.
