# Supplementary material for: Interaction of Listeria monocytogenes with the human choroid plexus endothelium in vitro: impact on invasion of the epithelium
Source: Hum Cell. 2026 Jul 10;39(7):104. doi: 10.1007/s13577-026-01419-8 (PMC13354639; doi:10.1007/s13577-026-01419-8)
Supplement: Supplementary file 2 — Supplementary file2 (PDF 424 KB) [file 13577_2026_1419_MOESM2_ESM.pdf]

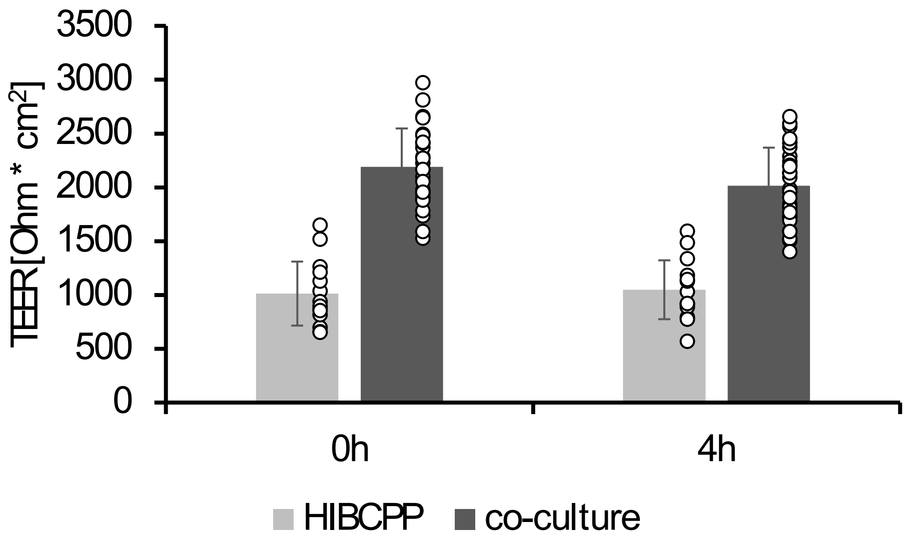

Fig. S2 TEER values of HIBCPP cells cultured in inverted culture system at the time of infection (0h) and during the experiments (4h). All experiments were performed at least three times in duplicates.
